# A chromosome-level assembly of the seed beetle *Callosobruchus maculatus* genome with annotation of its repetitive elements

**DOI:** 10.1093/g3journal/jkad266

**Published:** 2023-12-13

**Authors:** Göran Arnqvist, Ivar Westerberg, James Galbraith, Ahmed Sayadi, Douglas G Scofield, Remi-André Olsen, Elina Immonen, Franziska Bonath, Philip Ewels, Alexander Suh

**Affiliations:** Animal Ecology, Department of Ecology and Genetics, Uppsala University, Uppsala SE75236, Sweden; Systematic Biology, Department of Organismal Biology, Uppsala University, Uppsala SE75236, Sweden; Department of Ecology, Environment and Plant Sciences, Stockholm University, Stockholm SE10691, Sweden; School of Biological Sciences, University of Adelaide, Adelaide 5005, Australia; Faculty of Environment, Science and Economy, University of Exeter, Cornwall TR10 9FE, UK; Rheumatology, Department of Medical Sciences, Uppsala University, Uppsala SE75236, Sweden; Evolutionary Biology, Department of Ecology and Genetics, Uppsala University, Uppsala SE75236, Sweden; Uppsala Multidisciplinary Center for Advanced Computational Science, Uppsala University, Uppsala SE75236, Sweden; Science for Life Laboratory, Department of Biochemistry and Biophysics, Stockholm University, Stockholm SE10691, Sweden; Evolutionary Biology, Department of Ecology and Genetics, Uppsala University, Uppsala SE75236, Sweden; Science for Life Laboratory, Department of Molecular Biosciences, The Wenner-Gren Institute, Stockholm University, Stockholm SE10691, Sweden; Seqera Labs, Barcelona 08005, Spain; Systematic Biology, Department of Organismal Biology, Uppsala University, Uppsala SE75236, Sweden

**Keywords:** Chrysomelidae, chromosome conformation capture, X chromosome assembly, transposable elements, *Tc1-Mariner*

## Abstract

*Callosobruchus maculatus* is a major agricultural pest of legume crops worldwide and an established model system in ecology and evolution. Yet, current molecular biological resources for this species are limited. Here, we employ Hi-C sequencing to generate a greatly improved genome assembly and we annotate its repetitive elements in a dedicated in-depth effort where we manually curate and classify the most abundant unclassified repeat subfamilies. We present a scaffolded chromosome-level assembly, which is 1.01 Gb in total length with 86% being contained within the 9 autosomes and the X chromosome. Repetitive sequences accounted for 70% of the total assembly. DNA transposons covered 18% of the genome, with the most abundant superfamily being *Tc1-Mariner* (9.75% of the genome). This new chromosome-level genome assembly of *C. maculatus* will enable future genetic and evolutionary studies not only of this important species but of beetles more generally.

## Introduction

The introduction of long-read sequencing techniques has dramatically improved our ability to generate de novo genome assemblies. Yet, for large, repeat-rich, and structurally complex genomes, the resulting assemblies are still typically very fragmented, which restricts the utility of genome assemblies for certain types of analyses (Sedlazeck *et al*. 2018). In systems where linkage groups cannot easily be identified, chromosome conformation capture techniques such as Hi-C now offer dramatic improvement of assembly contiguity and scaffold length (Burton *et al*. 2013; Dudchenko *et al*. 2017). These techniques retain long-range genomic information, through crosslinking of chromatin and sequencing of proximal pairs of sequences, which can be used to construct chromosome spanning assemblies.

The seed beetle *Callosobruchus maculatus* (Coleoptera; Bruchinae) is a major agricultural pest on legume crops in arid regions of the world, causing crop losses of up to 90% (Sallam 2013), and is an established model system for studies in ecology and evolution (e.g. Holmes *et al*. 2020; Arnqvist *et al*. 2022). The recent publication of an annotated genome assembly of *C. maculatus* (Sayadi *et al*. 2019) now provides opportunities for novel use of this model also in genetics and genomics.

Here, our aim was 2-fold. First, the current assembly of *C. maculatus* is of high quality in terms of e.g. functional completeness and base accuracy but contains >15,000 contigs. We were interested in employing Hi-C to improve the contiguity by super-scaffolding the current assembly, ideally into chromosome-level scaffolds. This would, for example, enable detection of large structural variants, analyses of linked selection, genomic landscapes of divergence, and comparative studies of genome collinearity and structural orthology. Second, we made a dedicated effort to improve the annotation of repetitive elements in the genome of *C. maculatus*. The previous assembly is based on long-read PacBio sequence data, and a run with RepeatModeler followed by RepeatMasker identified a very high fraction of repeats in the assembly. However, more than half of these repeat sequences (54%) could not be attributed to any specific repeat class (Sayadi *et al*. 2019). This likely reflects rapid sequence evolution of repetitive elements in this group of insects, previously inferred from studies of variation in genome size (Arnqvist *et al*. 2015; Boman and Arnqvist 2023) and mitochondrial genomes (Sayadi *et al*. 2017). Our efforts aimed at increasing our understanding of the apparently rapid evolution of tandem repeats and transposable elements (TEs) and at alleviating the general underrepresentation of well-annotated beetles in repeat databases used for repeat classification (Parisot *et al*. 2021).

## Materials and methods

The genome of *C. maculatus* is ∼1.23 Gb (Arnqvist *et al*. 2015), and the estimated repeat content of the genome is as high as 71% (Sayadi *et al*. 2019). The karyotype of the genome is 2n = 18 + XX/XY, where the relative size of the 9 autosomes (% of the total haploid chromosomal length) ranges from 8.25 to 12.83, while the X is 7.69 and Y is a very small but distinct dot chromosome (Angus *et al*. 2011). We used the annotated *C. maculatus* reference genome assembly reported in Sayadi *et al*. (2019) as starting point for super-scaffolding and repeat annotation (GCA_900659725.1). In that work, PacBio long-read sequences representing 32× genome coverage with an average read length of 9.0 kb were assembled using FALCON and subsequently error-corrected based on realignment of both PacBio (32×) and Illumina (125×) reads. This assembly is 1.01 Gb in total size, with a contig N50 of 212 kb and the longest contig spanning 2.1 Mb. Annotation of the assembly was based on large amounts of transcriptome data, homology, and ab initio prediction methods and identified 21,264 coding genes. Analyses of conserved proteins sets showed a high fraction of well-assembled genes in the assembly (Sayadi *et al*. 2019). Yet, because of the high repeat content of the genome, the assembly is highly fragmented and contains >15,000 contigs.

### Hi-C library preparation, sequencing, and assembly

We used a sample of live male *C. maculatus* from the isogenic reference line SI4 for Hi-C sequencing. Beetles were killed by flash freezing in liquid nitrogen and were then ground in batches into a flour-like powder using a plastic pestle in an Eppendorf tube on dry ice. The pestle and tube used were precooled in liquid nitrogen prior to grounding, and the tube contained some liquid nitrogen, to ensure that the material remained frozen through the entire grinding, preparation, and transfer process. The sample was then stored at −80°C.

The Hi-C library was prepared following the Arima Protocol “Arima-HiC_AnimalTissue_v00” (document number A160126 v00). In short, 400 mg of ground beetle material was crosslinked in 2% formaldehyde for 20 min while rotating. The crosslinking was stopped by addition of the Arima-kit provided by Stop Solution. Before continuation, larger debris was allowed to sink to the bottom and only material small enough to pipette was used in subsequent reactions. Lysis was performed on crosslinked tissue equivalent to ∼2 μg of chromatin. All steps of the Hi-C reaction were performed as described in the Arima protocol.

The chromatin was fragmented in an AFA Fiber Crimp-Cap microTUBE using a COVARIS E220 with the following settings: peak incident power 175 W, acoustic duty factor 10%, 200 cycles per burst, and 50 s treatment time. The fragmented chromatin was purified using AMPure XP beads and subjected to library preparation following the “Arima-HiC Kit, Library Preparation using Illumina TruSeq DNA PCR-Free Library Prep” protocol (document number A160111 v01), and the Arima-kit reagents were supplemented by reagents of an Illumina TruSeq PCR-Free library preparation kit. The library was amplified for 11 cycles using an Illumina TruSeq DNA CD Index. The final library was analyzed for fragment length distribution using an Agilent Fragment Analyzer with a high-sensitivity NGS Fragment 1-6,000 bp kit and for concentration using the Qubit high-sensitivity dsDNA kit. Subsequently, the library was sequenced with a depth of ∼800 million reads on a NovaSeq 6000 S4 flow cell with a read length of 2 × 150 bp.

The Hi-C reads were preprocessed using Juicer (git-rev. 84f6957) (Durand *et al*. 2016) and a script provided by Arima Genomics to generate ligation site positions in the input assembly (GCA_900659725.1) matching the sequence motifs “GATCGATC,” “GANTGATC,” “GANTANTC,” and “GATCANTC.” The resulting list of valid Hi-C pairs was used as input for the scaffolder 3D-DNA (v. 180922). The resulting draft scaffolded assembly was manually error-corrected and curated using the JBAT method provided by the authors of 3D-DNA (Dudchenko *et al*. 2017).

In order to assess gene richness in major scaffolds and to identify scaffolds corresponding to the X chromosome, we mapped (1) all genes and (2) all putative X-linked genes identified in the original assembly from relative coverage in male and female samples (see Sayadi *et al*. 2019), using BWA-MEM (Li 2013), and assessed enrichment among scaffolds in the new assembly.

### Annotation liftover

The resulting Hi-C genome assembly was annotated by performing a coordinate conversion of the extant annotation [National Center for Biotechnology Information (NCBI), accession PRJEB30475]. We performed the liftover using Liftoff (Shumate and Salzberg 2021), using default parameters apart from a few that were set to more stringent criteria (coverage >80%, sequence identity >90%, distance scaling 5).

### Annotation of repetitive elements

A repeat library was built by manually curating part of the output of RepeatModeler open-1.0.11 (Smit *et al*. 2010). An initial repeat library was constructed using RepeatModeler and used to mask with RepeatMasker (Smit and Hubley 2010). To improve the annotation, manual curation of the 38 most abundant repeats which had already been classified by RepeatModeler as well as 89 of the most abundant repeats out of the total 490 classified as “unknown” was performed through a “BLAST-extend-align-trim” approach as previously described in Suh *et al*. (2018). Briefly, for each repeat subfamily consensus sequence identified by RepeatModeler, this approach consisted of (1) searching for copies of the subfamily using BLASTN (Altschul *et al*. 1990), (2) selecting the top 20 sequence hits, (3) extending their flanks, and (4) aligning the extended sequences using MAFFT (Katoh *et al*. 2002). For manual curation, consensus sequences were constructed using Advanced Consensus Maker (www.hiv.lanl.gov: Advanced Consensus Maker, last accessed 2019) after trimming of the discordant flanks. This new consensus was then classified using the CENSOR (Kohany *et al*. 2006) against the Repbase repeat database (Bao *et al*. 2015), NCBI's Conserved Domain Database search tool (Marchler-Bauer *et al*. 2017), and LAST alignment tool (Kiełbasa *et al*. 2011). Based on homology to known repeats, target site duplications (TSDs) in the alignment with flanks, presence of conserved protein domains, and self-alignment, repeat classification was determined in line with previous studies (Wicker *et al*. 2007; Feschotte and Pritham 2007). The resulting partially curated library was combined with a beetle-specific library from Repbase, created with RepeatMasker's queryRepeatDatabase.pl script (-species coleoptera), and then used to mask the genome using RepeatMasker.

## Results and discussion

### Hi-C assembly

**Fig. 1.**
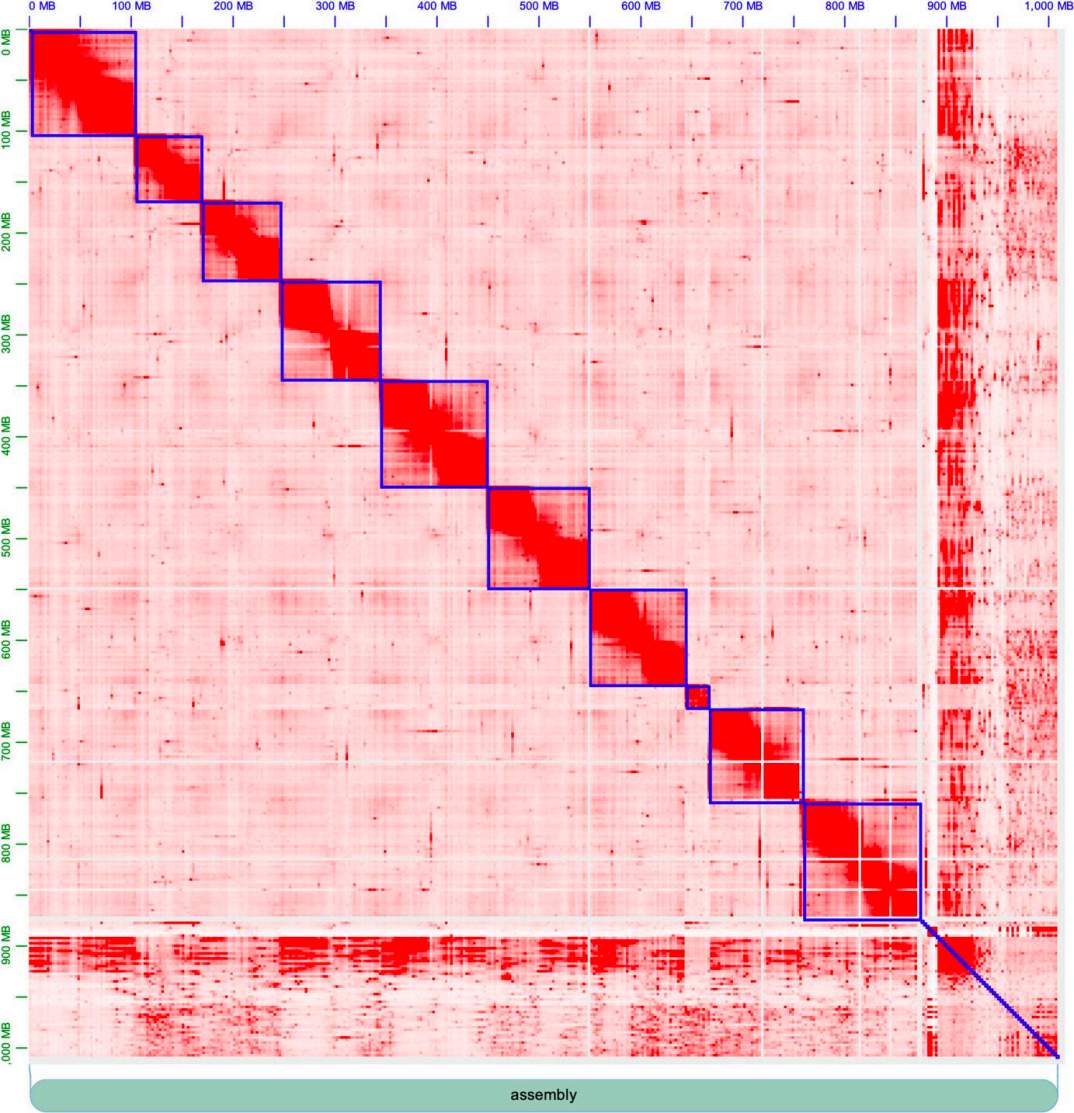
Hi-C contact map on the final *C. maculatus* assembly. Some 86% of the input assembly was arranged into 10 chromosome-length scaffolds.

**Fig. 2.**
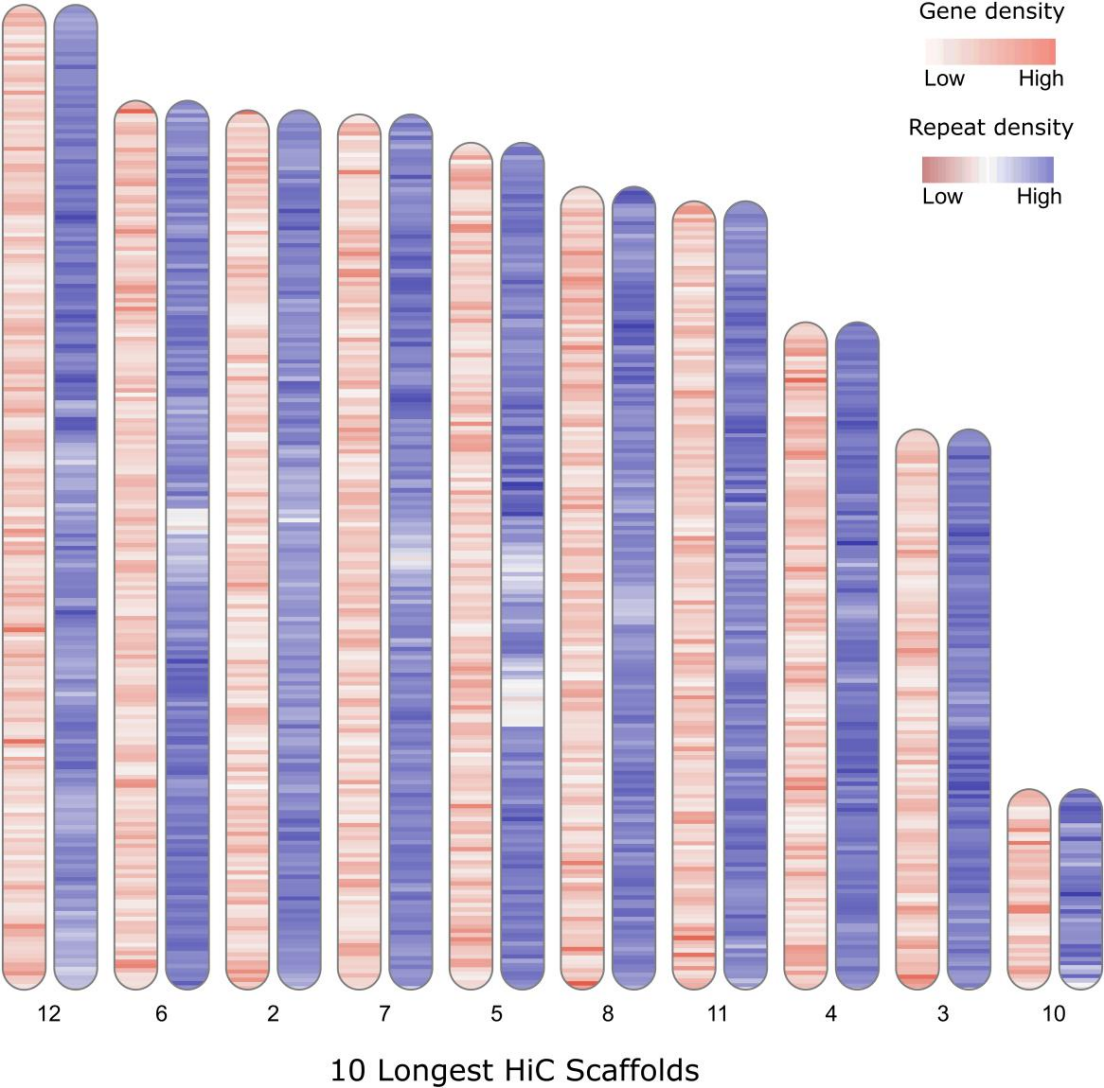
A chromosomal ideogram of gene and repeat densities within the 10 longest scaffolds of the *C. maculatus* Hi-C assembly. Densities were calculated in nonoverlapping 500 kbp bins. For scale, the length of the longest scaffold shown is 114.6 Mbp, and that of shortest is 23.3 Mbp.

After mapping to the input assembly, deduplicating and removing short fragment reads, Juicer outputted 141 M valid Hi-C contacts and additionally 154.7 M lower quality contacts [read-pairs below the mapping quality (MAPQ) threshold]. The resulting scaffolded assembly is 1.01 Gb in total size, which is on par with the predicted genome size of 0.96 Gb (Arnqvist *et al*. 2015), and it shows a massive improvement in scaffold length (see Table 1 for assembly statistics). In the end, 86% of the assembly is contained within 10 well-supported chromosome-length scaffolds (Fig. 1). The relative size and structure of the scaffolds is consistent with the 9 metacentric autosomes previously documented in *C. maculatus* using cytogenetics (Angus *et al*. 2011) with the single considerably smaller scaffold corresponding to a substantial fraction of the metacentric X chromosome. The identity of the X chromosome scaffold (23.3 Mb) was verified by the fact that 462 out of 658 putatively X-linked genes (Sayadi *et al*. 2019) mapped to this scaffold. We note that 81% of all genes and 83% of all coding sequences (CDSs) mapped to one of the 10 largest scaffolds. Gene density was variable along scaffolds, but no scaffold showed a general enrichment in gene content (Fig. 2).

**Table 1.** A comparison between the previous and the new genome assembly of *C. maculatus*.

	PacBio assembly (GCA_900659725.1)	New Hi-C assembly (CASHZR040000000.4)
Genome assembly size (Mbp)	1,007.82	1,012.33
No. of scaffolds/contigs	15,778	10,661
Maximum scaffold/contig length (Mbp)	2.07	114.627
Scaffold/contig L50	1,183	5
Scaffold/contig L90	6,491	472
Scaffold/contig N50 (bp)	212,245	98,582,428
Scaffold/contig N90 (bp)	26,507	39,277
Number of scaffolds/contigs >50 kbp:	4,364	245
% genome in scaffolds/contigs >50 kbp:	82.30%	88.98%
BUSCO assessment (*n* = 2124)		
Complete BUSCOs (C)	85.4% (*n* = 1816)	85.7% (*n* = 1820)
Duplicated BUSCOs (D)	4.8% (*n* = 103)	4.2% (*n* = 90)
Fragmented BUSCOs (F)	5.1% (*n* = 108)	5% (*n* = 107)

Completeness assessed with BUSCO v5.2.2, using the endopterygota_odb10 reference gene set.

All gene models in the liftover annotation carry additional attributes describing the coverage and identity statistics found during the liftover from the original annotation. A total of 36 gene models (out of 21,264) did not map to the scaffolded assembly, and a total of 744 gene models were partially mapped, using stringent criteria.

Our effort illustrates the great utility of Hi-C sequencing for super-scaffolding of complex and large genomes. The new and improved genome assembly of *C. maculatus* will no doubt aid in the control of this widespread agricultural pest and should also significantly increase the utility of this model species in future genomic and genetic studies. For example, with chromosome-level assemblies now being available for a growing number of beetle species (e.g. Herndon *et al*. 2020; Zhang *et al*. 2020; Chen *et al*. 2021; Keeling *et al*. 2022), future studies of shared synteny and genome collinearity promises insights into the evolution of genome structure in this large group of insects. Further, information on physical colocalization of genes and contigs will enable analyses of the role of linkage and linked selection in evolutionary genomic studies that utilize experimental evolution or artificial selection and can provide detailed insights into the genomic landscape of population and species divergence.

### Repetitive elements

**Fig. 3.**
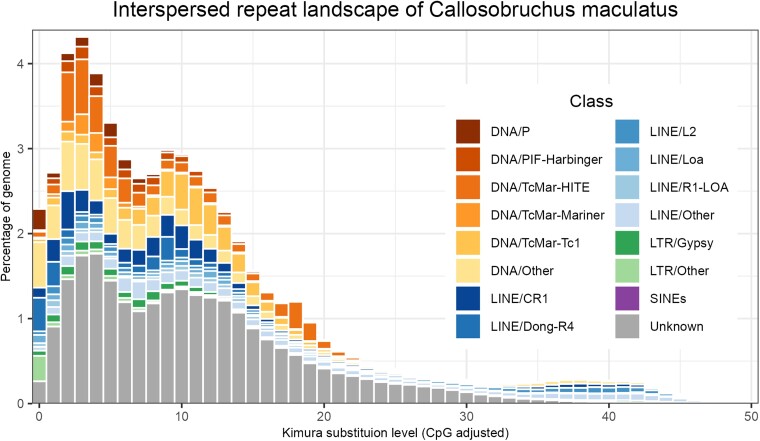
Repeat landscape of the *C. maculatus* genome. The X-axis shows the Kimura 2-parameter distance of repeat copies to their respective consensus sequence, with low values indicating that the repeat copy is more recent. The Y-axis shows the cumulative genome percentages of the repeats in each 1% bin of Kimura substitution level. The colored parts of the bars correspond to different classifications of repeats with gray being the unclassified repeats (“unknown”).

Both before and after automated classification and manual curation of the most abundant repeats, the total masked repeat content accounted for 70% percent of the assembly (Fig. 3). Through our manual curation, we were able to classify 83 of the 89 most common unclassified repeat subfamilies: 67 as DNA transposons, 7 as long interspersed nuclear elements (LINEs), 7 as LTR retrotransposons, and 2 as satellite DNA. This greatly reduced the proportion of repetitive elements which were unclassified from 31% of the assembly to 24%. Of all repeats with known classification annotated by RepeatMasker, DNA transposons were the most abundant, covering 18% of the genome, followed by LINEs (13%) and LTR elements (2.3%).

**Fig. 4.**
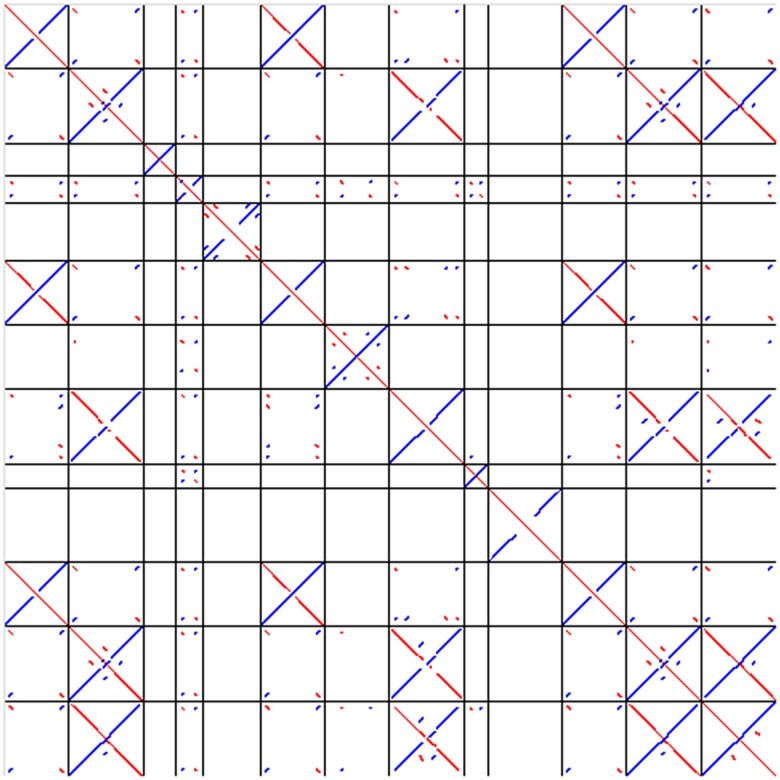
Dot plot of consensus sequences of the 13 newly identified large nonautonomous *Tc1-Mariner* DNA transposons when aligned to each other using LAST. The lengths of the consensus sequences range from 654 to 2,199 bp.

The most abundant superfamily was *Tc1-Mariner*, within the class of DNA transposons, accounting for 9.75% of the genome. A large portion of the annotated Tc1-Mariner elements (5.2% of the genome, 41.7% of *Tc1-Mariner* elements) were of a group of large (1–2 kb) nonautonomous elements similar to miniature inverted-repeat TEs (MITEs), possessing large terminal inverted repeats (TIRs), often being fully inverted and essentially forming large palindromes (Fig. 4).

The genomes of a few other leaf beetles (Chrysomelidae) have now been assembled, with larger assembly sizes not obviously correlating to repeat content. Genomes of *Leptinotarsa* species were found to harbor 27–34% repeats and are 512–643 Mb in size (Cohen *et al*. 2021), *Galerucella* species 41–49% and 460–588 Mb (Yang *et al*. 2021), *Ophraella communa* 58.2% and 774 Mb (Bouchemousse *et al*. 2020), *Gonioctena quinquepunctata* 66% and 1.73 Gb (Lukicheva *et al*. 2021), *Diabrotica balteata* 47% and 1.61 Gb (King *et al*. 2023), *Diabrotica virgifera* 53% and 1.85 Gb (Coates *et al*. 2023), and *Altica viridicyanea* 63% and 865 Mb (Xue *et al*. 2021). However, different sequencing technologies were used across these taxa, which will impact the portion of the repeat content of the genomes sequenced, assembled, and annotated (Peona *et al*. 2021). The long-read data that forms the basis for the *C. maculatus* assembly likely contributes to the higher repeat content relative to assembly size compared with other leaf beetles. We note that the repeat content varied strikingly along scaffolds but no scaffold showed an obvious enrichment in repeats (Fig. 2).

A large portion of the repeat content of *C. maculatus* was *Tc1-Mariner* DNA transposons. Of these, many were nonautonomous and palindromic, similar to MITE transposons in plants (Feng *et al*. 2002; Feschotte *et al*. 2002). However, the length of these MITE-like *Tc1-Mariner* consensus sequences ranges up to 2.2 kb, which is much larger than the typical size of MITEs (a few hundred bp; Ye *et al*. 2016). The full extent of their impact on the overall genome evolution, for example through their potential for cut-and-paste transposition, will need comparisons with other species. However, it is clear that these large palindromic TEs make up a significant portion of the *C. maculatus* repetitive landscape. In closing, we note that seed beetles may also provide future insights into the possible role of repeats in adaptive evolution, as genome size is associated with organismal function (Arnqvist *et al*. 2015; Boman and Arnqvist 2023) and because transcripts related to DNA-mediated transposition show differential abundance in experimental life history evolution lines in seed beetles (Immonen *et al*. 2023).

## Data Availability

Preprocessing scripts are available at https://github.com/ArimaGenomics/Scripts. Data, assembly, and annotation files have been deposited at the European Nucleotide Archive (ENA) under the BioProject PRJEB60338 as accession CASHZR040000000. The repeat library has been deposited at the Zenodo repository and is available at https://zenodo.org/record/7994921. A file containing the genomic locations of repeats, as well as the repeat family to which each identified repeat belongs, has been deposited at Mendeley Data and is available at https://data.mendeley.com/datasets/6w6h63nw4s/2.
